# A Multifeatures Fusion and Discrete Firefly Optimization Method for Prediction of Protein Tyrosine Sulfation Residues

**DOI:** 10.1155/2016/8151509

**Published:** 2016-03-10

**Authors:** Song Guo, Chunhua Liu, Peng Zhou, Yanling Li

**Affiliations:** ^1^School of Computer and Information Technology, Xinyang Normal University, Xinyang 464000, China; ^2^College of Information Engineering, Tarim University, Alar, Xinjiang 843300, China; ^3^Department of Electronic and Communication Engineering, Zhengzhou Institute of Aeronautical Industry Management, Zhengzhou 450015, China

## Abstract

Tyrosine sulfation is one of the ubiquitous protein posttranslational modifications, where some sulfate groups are added to the tyrosine residues. It plays significant roles in various physiological processes in eukaryotic cells. To explore the molecular mechanism of tyrosine sulfation, one of the prerequisites is to correctly identify possible protein tyrosine sulfation residues. In this paper, a novel method was presented to predict protein tyrosine sulfation residues from primary sequences. By means of informative feature construction and elaborate feature selection and parameter optimization scheme, the proposed predictor achieved promising results and outperformed many other state-of-the-art predictors. Using the optimal features subset, the proposed method achieved mean MCC of 94.41% on the benchmark dataset, and a MCC of 90.09% on the independent dataset. The experimental performance indicated that our new proposed method could be effective in identifying the important protein posttranslational modifications and the feature selection scheme would be powerful in protein functional residues prediction research fields.

## 1. Introduction

Tyrosine sulfation is one of the most prevalent posttranslational modifications in transmembrane and secreted proteins. Many lines of evidence have suggested that nearly 1% of all tyrosine residues of the total proteins in an organism can be sulfated [1]. Tyrosine sulfation has been found to be participating in the interactions between proteins and the modulations of intracellular proteins [2, 3]. Malfunction or dysregulation of tyrosine sulfation would lead to several serious diseases, such as atherosclerosis [4], lung diseases [5], and HIV infections [6]. Therefore, identification of possible protein tyrosine sulfation substrates and their accurate residues is valuable in exploring the intrinsic mechanism of tyrosine sulfation in biological processes and therefore arouses interests of biologists in these fields.

In view of the laborintensive and time-consuming biochemical experiments, computational intelligence technologies are becoming more and more popular due to their conveniences as well as efficiencies. In the past decades, many computational methods have been proposed and successfully applied in this field [7–14].

In 1997, Bundgaard et al. made the first attempt to predict the tyrosine sulfation residues based on sequence comparisons by using synthetic peptides [7]. They pointed out that the tyrosylprotein sulfotransferase was cell-specifically expressed. In 2002, Monigatti et al. constructed the first software tool named Sulfinator based on four different hidden Markov models to identify tyrosine sulfation residues [8]. Yu et al. developed a log-odds position-specific scoring matrix (PSSM) to construct the prediction model [9]. They found that tyrosine sulfation residues mostly located in extracellular tail and extracellular loop 2. Subsequently, Monigatti et al. gave an overview of sulfation in the context of modificomics [10]. Chang et al. proposed a computational method named SulfoSite based on support vector machine (SVM) [11]. Niu et al. developed a method by using maximum relevance minimum redundancy (mRMR) method to select the best feature subset and nearest neighbor algorithm to construct the predictor [12]. PredSulSite introduced two new encoding schemes, namely, grouped weight and autocorrelation function [13]. Jia et al. proposed a novel method named SulfoTyrP by using undersampling approach and weighted support vector machine [14].

All abovementioned methods facilitated the investigations on tyrosine sulfation; however, the accuracy was still far from satisfactory and detailed analyses of the features are lacking. Thus, it was significant to develop a powerful predictor to identify the tyrosine sulfation residues.

In this paper, we focused on the challenging problem of predicting tyrosine sulfation residues based on protein sequences. Firstly, several informative sequence-derived features were combined to construct the feature vector. Secondly, relative entropy selection and incremental feature selection (RES + IFS) were adopted to perform the preevaluation of the features, and then discrete firefly algorithm (DFA) and SVM were introduced to perform the second-round feature selection as well as build the predicted model. Experimental results on the benchmark datasets and independent datasets proved that our method was a powerful tool for tyrosine sulfation residues prediction. A web-server of DFA_PTSs was constructed and freely accessible at http://biolabxynu.zicp.net:9090/DFA_PTSs/ for academic use.

## 2. Materials and Methods

### 2.1. Datasets

To reach a consensus assessment with previous researches [8, 12, 13], two benchmark datasets were collected in this work. The datasets were compiled from UniProtKB (2013.09) [15] with the keyword “sulfotyrosine.” Then, these proteins were clustered with no more than 30% similarities by CD-HIT [16]. Finally, 137 experimental tyrosine sulfation residues from 79 protein sequences were collected. 68 proteins (119 tyrosine sulfation residues) were selected as a training set and the remaining 11 proteins (18 tyrosine sulfation residues) were selected as an independent test.

The peptide segments of tyrosine sulfation residues and non-tyrosine sulfation residues could be formulated by

(1) $$\begin{array}{c} P=R_{-\xi }R_{-\left(\xi -1\right)}\cdots R_{-2}R_{-1}YR_{+1}R_{+2}{\cdots R}_{+\left(\xi -1\right)}R_{+\xi }, \end{array}$$

where *ξ* indicated the distance between the furthest residue and the target residue and 2*ξ* + 1 would be the sliding window length. In order to test the proposed model fairly as well as keep consistent with previous studies [8, 12, 13], *ξ* was set as 4 and the corresponding sliding window length would be 9. However, in some cases, the upstream or downstream number of residues for a tyrosine may be less than 4. The lacking residues would be filled with dummy code *X*.

### 2.2. Feature Encoding

#### 2.2.1. PSI-BLAST-Based Features

As is well known, the life originated from ancient limited peptides. With the development of evolution and nature selection, various sequences began to appear and form the complex organisms. In the process of sequence evolution, some unimportant peptides disappeared while the important function-determinate regions were kept. Considering this, evolutionary conservation had been widely used to explore the attributes of proteins, such as predicting the extracellular matrix proteins [17] and identifying the epitopes [18] and cysteine S-nitrosylation residues [19].

To obtain evolutional conservation profiles, PSSM was generated by the program PSI-BLAST [20] with default parameters (3 iterations and 0.0001 of *E*-value cutoff) against the Swiss-Prot database (http://www.ebi.ac.uk/swissprot/). The evolution conservation for a protein *P* with *L* residues would be given as the following matrix:

(2) $$\begin{array}{c} P_{PSSM}=\left[\begin{array}{cccc} S_{1\to A} & S_{1\to R} & \cdots & S_{1\to V}\\ S_{2\to A} & S_{2\to R} & \cdots & S_{2\to V}\\ \vdots & \vdots & \cdots & \vdots \\ S_{L\to A} & S_{L\to R} & \cdots & S_{L\to V} \end{array}\right], \end{array}$$

where *S*_*i*→*j*_, *i* = 1,2, 3,…, *L*, represented the frequency of the *i*th position residues which was substituted by amino acid *j* (*j* = 1,2, 3,…, 20) in the evolution history. The positive scores indicated that this substitution appeared more frequently than that expected, while the negative scores meant the opposite. Usually, the aggregation of positive scores indicated the important function zones in the proteins. Considering this, to make the descriptor uniformly cover the peptides, elements in the above equation for PSSM were used to define a new matrix *M*_PSSM_, which was formulated by

(3) $$\begin{array}{c} M_{PSSM}=\left[\begin{array}{cccc} \sum S_{A\to A} & \sum S_{R\to A} & \cdots & \sum S_{V\to A}\\ \sum S_{A\to R} & \sum S_{R\to R} & \cdots & \sum S_{V\to R}\\ \vdots & \vdots & \cdots & \vdots \\ \sum S_{A\to V} & \sum S_{R\to V} & \cdots & \sum S_{V\to V} \end{array}\right], \end{array}$$

where ∑*S*_*i*→*j*_ indicated the sum of amino acids type *i* being changed to amino acids type *j* in *P*_PSSM_. Finally, 400 features were obtained to describe the evolutionary conservation of the adjacent regions of the tyrosine sulfation residues.

#### 2.2.2. PSIPRED-Based Features

Previous researches figured out that the proteins with the same structural class but low sequence similarity may still keep some attributes in their secondary structure. Hence, in this paper, the information of secondary structure was adopted for identifying the tyrosine sulfation residues. PSIPRED [21], which applies two-stage neural networks to predict secondary structures, has found wide applications in computational biology, such as solvent accessibility [22], epitope recognition [18], cysteine S-nitrosylation sites [19], and protein folding kinetic types [23]. According to [21], the output files of PSIPRED were encoded with terms of “*C*” for coil, “*H*” for helix, and “*E*” for strand. Here, we quantified the total number, average length, and percentage of each peptide, which were defined as follows:

(4) T_numα=∑α,Ave_lenα=∑α∑peptideα,Com_perα=∑α∑H+∑E+∑C×100%,

where *α* = {*H*, *E*, *C*}. Finally, 9 features were derived to construct the predicted secondary structure features.

#### 2.2.3. Native Disorder Features

Natively disordered zone has been proved to be connected with many various physiological activities, such as epitope recognition, solvent accessibility, and protein interaction [18, 24, 25]. Hence, they were often used in researches of protein structures and functions. Here, DISOPRED [26] was used to predict the disorder status for each residue in the peptides. In summary, 9 features were obtained to construct the native disorder features.

#### 2.2.4. Protein Physicochemical Features

As is well known, the hydrophobic residues tended to form small patches on the surface of the proteins to participate in the interaction. Some residues with polarity and charge could play a critical role in protein binding [22]. In addition, the flexibility and accessibility of a residue strongly affected the protein functional residues. Therefore, in this work, 6 physicochemical properties (hydrophilicity, flexibility, accessibility, polarity, exposed surface, and turns) were collected to predict protein tyrosine sulfation residues.

### 2.3. Discrete Firefly Optimization Algorithm

The firefly algorithm (FA) [27] is a novel heuristic optimization algorithm inspired by the natural behaviors of fireflies. FA has been proved to be a very effective optimization algorithm to search the global optima. The DFA is the modified traditional firefly algorithm which could be used in solving discrete optimization problems. The pseudocode of the DFA was shown in Procedure 1.

**Procedure 1: Pseudocode of the DFA.**
**Begin**
   
**Input**: firefly population *X*_*i*_ (*i* = 1,2,…, *n*), lightness *L*_*i*_; light absorption coefficient *γ*, MaxGeneration MG.
   
**While** (*t* < MG)
    
**For** *i* = 1 : *n*
    
*  * 
**For** *j* = 1 : *i*
     
*  *  
**If** (*L*_*j*_ > *L*_*i*_),
          move firefly *i* towards *j*;
     
*  *  
**Else**
     
*  *  Attractiveness varies with distance *r* via *e* ^−*γr*^
     
*  *  
**End if**
     
*  *  Evaluate new populations & update lightness
    
*  * 
**End for**
    
**End for**
    Find the current best firefly
   
**End while**
   
**Output**: the global best firefly(solution)
**End**

*Distance.* The distance between any two fireflies *f*_*i*_ and *f*_*j*_ was defined as follows:

(5) $$\begin{array}{c} r_{ij}=\left\|\;x_{i}-x_{j}\;\right\|=\sqrt{\overset{d}{\underset{k=1}{\sum }}{\left(x_{i,k}-x_{j,k}\right)}^{2}}, \end{array}$$

where *x*_*i*,*k*_ was the *k*th component of the *i*th firefly.

*Attractiveness.* The attractiveness of a firefly was determined by its lightness, which implied how strong it attracted the adjacent fireflies:

(6) $$\begin{array}{c} \beta \left(\;r\;\right)=\beta_{0}e^{-\gamma r^{m}},\;m\geq 1, \end{array}$$

where *r* was the distance between two fireflies, *β*_0_ was the attractiveness, and *γ* was a fixed light absorption coefficient.

*Movement.* The movement of a firefly was determined by the attractiveness from other fireflies. It was formulated as

(7) $$\begin{array}{c} X_{i}=X_{i}+\beta \times e^{-\gamma r_{ij}^{2}}\left(\;X_{j}-X_{i}\;\right)-\alpha \times \left(\;\mathrm{rand}-\frac{1}{2}\;\right). \end{array}$$

*Discretization.* If firefly *i* moved toward *j*, the position of firefly *i* changed from a binary number to a real number. In this study, the sigmoid function was used to constrain the position value to the interval [0,1]:

(8) $$\begin{array}{c} S\left(\;x_{ik}\;\right)=\frac{1}{1+e^{-x_{ik}}}, \end{array}$$

where *S*(*x*_*ik*_) indicated the probability of *x*_*ik*_.

*Fitness Definition.* In this paper, the prediction accuracy and the number of selected features were the two criteria to design a fitness function. Therefore, the fitness function had two predefined weights, *w*_*α*_ for the prediction accuracy (in this paper, we chose the MCC) and *w*_*β*_ for the selected features, which were formulated as follows:

(9) $$\begin{array}{c} {fit}_{i}=w_{\alpha }\times {MCC}_{i}+w_{\beta }\times \left[\;1-\frac{\left(\sum_{1}^{n}i\right)}{n}\;\right]. \end{array}$$

### 2.4. Relative Entropy Selection and Incremental Feature Selection

Although the combination of different types of features would provide a more powerful predictor, some unwanted noise features which were called “bad” features may also be brought in at the same time. These unwanted noise features may decrease the prediction and generalization performance of the classifiers. To reject the bad features as well as keep the good features, we here adopted relative entropy selection (RES) (i.e., Kullback-Leibler divergence) [28] to select the optimal feature subset. For the algorithm, relative entropy was defined as follows:

(10) $$\begin{array}{c} DKL\left(\;P||Q\;\right)+DKL\left(\;Q||P\;\right), \end{array}$$

where *P* and *Q* were the conditional probability density functions of a feature under two various categories; DKL(*P*||*Q*) was the K-L divergence of *Q* from *P* and DKL(*Q*||*P*) was the K-L divergence of *P* from *Q* [19]. A feature list *L* would be obtained after the relative entropy selection:

(11) $$\begin{array}{c} L=\left\{\;f_{1},f_{2},f_{3},\ldots ,f_{i},\ldots \;\right\},\;i\in \left\{1,2,3,\ldots ,N\right\}, \end{array}$$

where the index *i* indicated the importance of *f*_*i*_ in the feature list *L*.

Once the ranked feature list was obtained, the incremental feature selection (IFS) procedure was used to search for the optimal feature subset for the predictor. During the IFS, the features in the list *L* would be added one by one from the head to the tail. In each iteration, a new feature would be added and form a new feature subset. For each new feature subset, we built a new classifier using 10-fold cross-validation. Then, 472 individual classifiers would be obtained for the 472 feature subsets. As a result, a table named IFS, with one column for the feature index and the other columns for the prediction performance, was produced. The IFS curve was drawn based on the IFS list to identify the best prediction efficiency as well as the corresponding optimal feature subsets.

### 2.5. Support Vector Machine

In statistical prediction, three cross-validations, namely, independent test, subsampling (*k*-fold cross-validation) test, and jackknife test, are often adopted to assess the performance of a predictor. In order to remain consistent with [8, 12, 13], 10-fold cross-validation was used to assess the proposed method. The benchmark dataset was initially randomly divided into 10 equal subsets. In each iteration, nine subsets were used for training and the remaining one was used for testing. The procedure would be repeated 10 times and the final results were calculated by averaging the 10 testing results.

**Figure 1:**
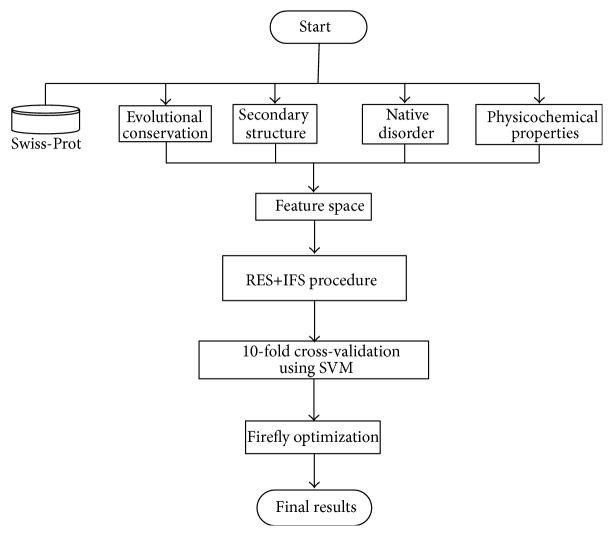
The system architecture of the proposed model.

Support vector machine (SVM) was a successful supervised learning method which found extensive use in classification and regression problems. In this work, LibSVM [29] was adopted to perform all the experiments. The system architecture of the proposed model was illustrated in Figure 1.

### 2.6. Assessment of Prediction Accuracy

Five routinely used assessment criteria were adopted here, that is, sensitivity (SN), specificity (SP), accuracy (ACC), Mathews correlation coefficient (MCC), and AUC (area under Receiver Operating Characteristic curve):

(12) SN=TPTP+FN,SP=TNTN+FP,ACC=TP+TNTP+FP+TN+FN,MCC=TP×TN−FP×FNTP+FNTP+FPTN+FPTN+FN,

where TP, TN, FP, and FN were the abbreviations of true positives, true negatives, false positives, and false negatives. In this paper, MCC was used as the major evaluation index to evaluate the performance of the new proposed predictor. The ROC (Receiver Operating Characteristic) curve was to plot the true positive rate against false positive rate, and the AUC was a reliable measure for evaluating performance.

## 3. Results and Discussion

### 3.1. Preevaluation of the Features

**Figure 2:**
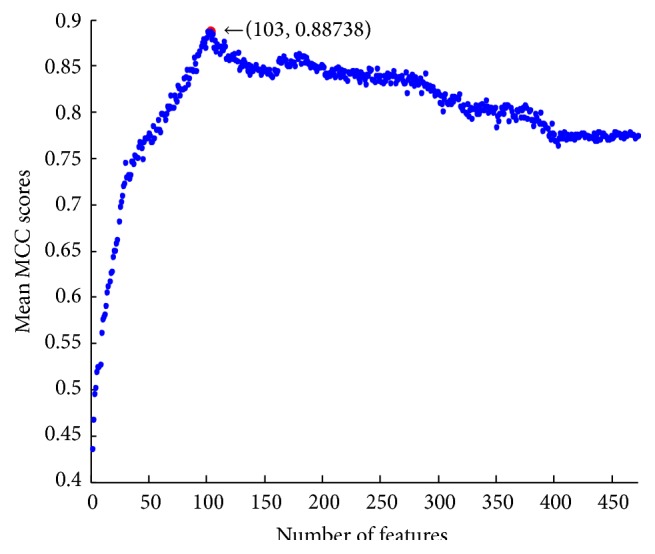
IFS scatter plot for 472 features.

After finishing the relative entropy selection, two lists, one called coefficient value list and the other called feature list, were obtained. In the relative entropy feature lists, a feature with a bigger coefficient index indicated that it is more important for predicting tyrosine sulfation residues. Subsequently, 472 predictors were built one after another by adding features one by one from the top of the list to the bottom. The mean MCC value for each predictor was given in Figure 2. When 103 features were given, the mean MCC values reach the peak value of 0.88738.

### 3.2. Features Selection and Parameters Optimization

In this work, we used RES + IFS to perform preevaluation of initial feature set and DFA to perform feature selection and parameters optimization. To evaluate the performance of this scheme, we compared our method with minimum Redundancy Maximum Relevance together with incremental feature selection (mRMR + IFS) in the preevaluation procedure and genetic algorithm (GA) [30] and discrete particle swarm optimization (DPSO) [31] in the second-round feature selection procedure. The experiments of RES + IFS and mRMR + IFS would use grid search to search parameters. GA, DPSO, and DFA would use the preselected 103 features obtained from RES + IFS to perform the second-round feature selection procedure. The parameter configurations were listed in Table 1.

**Figure 3:**
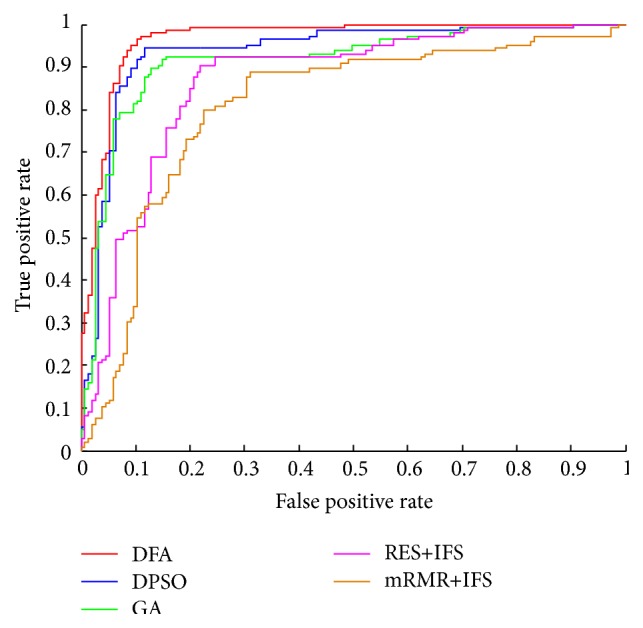
The ROC curve of four algorithms.

The experimental results were given in Table 2 and Figure 3. RES + IFS selected 103 features and gave a MCC of 88.74%, while the mRMR + IFS produced a MCC of 84.65% based on 127 features. In addition, RES + IFS was much faster than mRMR + IFS. Thus, we choose RES + IFS procedure to perform the preevaluation of features. The GA algorithm obtained a MCC of 91.69% and an AUC of 88.33% and selected an optimal feature subset of 73 features. The DPSO algorithm produced a slight improvement of a MCC of 92.66% and an AUC of 91.79% while it selected the least 62 features. Generally, the DFA performed the best among these three optimization algorithms (a MCC of 94.41% and an AUC of 92.45%). Although DFA selected 3 more features than DPSO did, it produced the highest MCC of 94.41%. Actually, DFA used the least computational time to converge. Thus, in this work, the DFA was chosen as the final optimization algorithm.

### 3.3. Analysis of the Optimal Feature Subset

**Figure 4:**
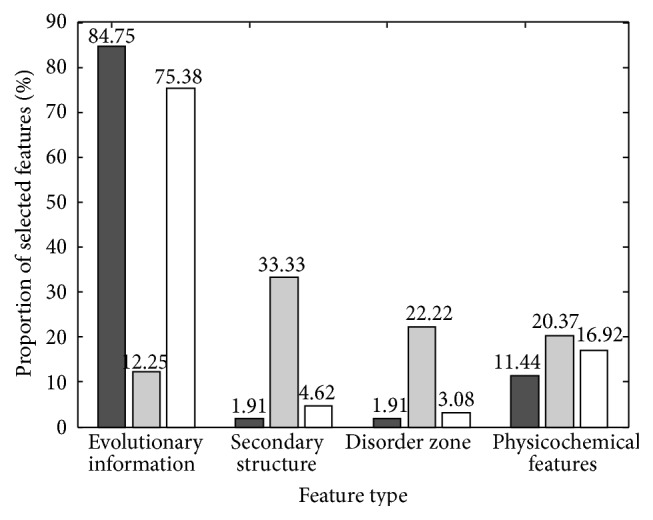
Various contributions of different features. The black bars indicated the proportion of the feature in the whole feature matrix; the grey ones represented the percentage of the selected features accounting for the corresponding feature type; and the white ones represented the percentage of the selected features accounting for the final optimal feature subsets.

In this part, we analyzed the final optimal feature subset in detail and investigated the various contributions of different features.  Figure 4 displayed the various contributions of different types of features. Among the 65 best features, 49 pertained to the evolutionary conservation, 3 to the secondary structure, 2 to the native disorder, and 11 to the physicochemical properties.

Obviously, evolutionary conservation occupied the largest part in prediction of tyrosine sulfation residues. As is known to all, various biological species originated from the limited peptides in ancient oceans. Evolution and selection existed in the whole story of life. The evolution in protein includes the mutations, insertions, and deletions of a single residue or some peptides. With the accumulation of time, some unimportant zone may disappear, but the functional regions may remain because they always share some common attributes. This explains why evolutionary conservation played the most important role in the optimal subset.

Although only 3 and 2 features were selected from the secondary structure and native disorder, respectively, one could not regard that the secondary structure and native disorder played less important roles in identifying the tyrosine sulfation residues. Actually, nearly 84.75% of features were from the evolution conservation, and only 1.91% of features were from the secondary structure and native disorder. In addition, almost 33.33% and 22.22% among the secondary structure and native disorder were selected in the optimal feature subset. Listed in Supporting Information S1 (see Supplementary Material available online at http://dx.doi.org/10.1155/2016/8151509) were the selected features.

### 3.4. Comparison with Other Methods

Listed in Table 3 were the experimental results performed by state-of-the-art methods on the independent dataset. Sulfinator [8] used sequence alignment; SulfoSite [12] used solvent accessibility area and maximum weight algorithm; and PredSulSite [13] used secondary structure, grouped weight, and autocorrelation function to construct the training features, respectively. In this paper, we adopted various informative sequence-derived features, namely, evolutional conservation, secondary structure, native disorder and physicochemical properties, and DFA algorithm and SVM, to construct the predicted model. Overall, our method exhibited the best prediction performance.

The excellent performance could be ascribed to two aspects: (i) the informative features, which included evolutional conservation, secondary structure, native disorder, and physicochemical properties (these features have been proven to be able to successfully distinguish the tyrosine sulfation residues from nonsulfation residues), and (ii) the powerful feature selection and parameter optimization method (this method included the preevaluation of the features using RES + IFS procedure and the second-round feature selection together with parameters optimization by using DFA).

### 3.5. Web-Server of DFA_PTSs

DFA_PTSs has been constructed and deployed as a free available web-server at http://biolabxynu.zicp.net:9090/DFA_PTSs/. Here, we provided a step-by-step guide for biology experimental scientists.

**Step 1 .**

**Figure 5:**
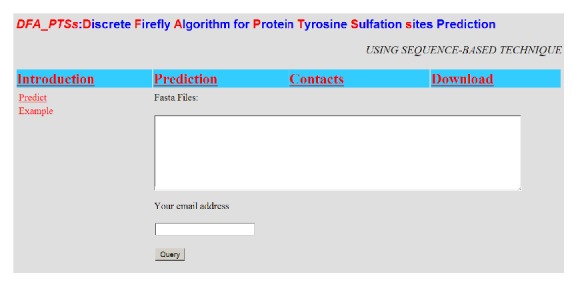
The home page of DFA_PTSs.

Open the web-server and you will find the home page (Figure 5). Click on the “Introduction” link to see a detailed description about the server.

**Step 2 .**

Either type or copy and paste the query protein sequences into the input box. DFA_PTSs accepts both single or multiple sequences input, which accords with standard FASTA format.

**Step 3 .**

Type your email address, and the predicted results will be sent to your email after finishing calculation.

**Step 4 .**

Click on the Query button to submit the request. In general, it takes no more than 2 minutes for a protein sequence with less than 300 amino acids.

## 4. Conclusions

In this paper, we presented a novel method to identify protein tyrosine sulfation residues. The proposed predictor achieved promising results and outperformed many other state-of-the-art predictors. The excellent performance should be ascribed to two aspects. The first aspect was the introduction of the informative features. These features included evolutional conservation, secondary structure, native disorder, and physicochemical properties. The second was the effectiveness of elaborate feature selection and parameter optimization schemes. This scheme included two procedures, namely, preevaluation of the features using RES + IFS procedure and the second round of feature selection using DFA. Finally, an optimal set of 67 features, which significantly contributed to the identification of tyrosine sulfation residues, were selected. Our predictor achieved the mean MCC of 94.41% on the benchmark dataset using 10-fold cross-validation, and a MCC of 90.09% on the independent dataset. The experimental performance indicated that our new proposed method could be useful in assisting the discovery of important protein modifications and the feature selection scheme would be powerful in protein function and structure prediction research domains.

## Supplementary Material

Supplementary Material: Detailed descriptions for the optimal feature subset. Among the 65 best features: 49 pertained to the evolutionary conservation, 3 to the secondary structure, 2 to the native disorder and 11 to the physicochemical properties.

## Figures and Tables

**Table 1:** The parameter configuration used in four optimization algorithms.

	Parameter configurations	
Grid search	[*c*min, *c*max]	[−5, 15]
	[*g*min, *g*max]	[−15, 5]

GA	Crossover	0.6
	Populations	20
	Mutation	0.033
	Max generation	1000

DPSO	Particles	100
	C1	1
	C2	2
	Max generation	1000

DFA	Group	100
	Randomness	0.9
	Absorption coefficient	0.5
	Max generation	1000

**Table 2:** The prediction performance of four algorithms.

	SN (%)	SP (%)	ACC (%)	MCC (%)	Features
RES+IFS^1^	91.49	96.01	94.67	88.74	103
mRMR+IFS^2^	86.71	91.66	90.08	84.65	127
GA^3^	92.55	97.17	94.28	91.69	73
DPSO^4^	93.73	97.59	95.04	92.66	62
DFA^5^	**95.37**	**98.67**	**97.41**	**94.41**	**65**

^1^ *C* = 64, *γ* = 0.03125 using Gauss kernel function; ^2^ *C* = 64, *γ* = 0.04268 using Gauss kernel function; ^3^ *C* = 128, γ = 0.003790 using Gauss kernel function; ^4^ *C* = 128, γ = 0.01136 using Gauss kernel function; ^5^ *C* = 128, γ = 0.005062 using Gauss kernel function.

**Table 3:** Comparisons of the proposed method with other methods.

	SN (%)	SP (%)	ACC (%)	MCC (%)
Sulfinator [8]	44.44	87.50	74.14	35.44
SulfoSite [12]	83.33	87.50	86.21	68.94
PredSulSite [13]	89.89	97.50	94.83	87.80
This method	**93.33**	**97.50**	**96.66**	**90.09**
