# Supplementary material for: A Multifeatures Fusion and Discrete Firefly Optimization Method for Prediction of Protein Tyrosine Sulfation Residues
Source: Biomed Res Int. 2016 Mar 10;2016:8151509. doi: 10.1155/2016/8151509 (PMC4806266; doi:10.1155/2016/8151509)
Supplement: Supplementary file 1 — Supplementary Material: Detailed descriptions for the optimal feature subset. Among the 65 best features: 49 pertained to the evolutionary conservation, 3 to the secondary structure, 2 to the native disorder and 11 to the physicochemical properties. [file 8151509.f1.docx]

Supporting Information S1

Detailed descriptions for the optimal feature subset

Among the 65 best features:

49 pertained to the evolutionary conservation (shown in red)

3 to the secondary structure (shown in blue)

2 to the native disorder (shown in green)

11 to the physicochemical properties (shown in black)

| 1 | **E/I** |
| --- | --- |
| 2 | **M/L** |
| 3 | **hydrophilicity(+1)** |
| 4 | **polarity(0)** |
| 5 | **C/V** |
| 6 | **F/R** |
| 7 | **P/F** |
| 8 | **flexibility(-3)** |
| 9 | **M/D** |
| 10 | **L/Q** |
| 11 | **E/F** |
| 12 | **hydrophilicity(-2)** |
| 13 | **S/D** |
| 14 | **G/L** |
| 15 | **G/T** |
| 16 | **D/P** |
| 17 | **accessibility(-2)** |
| 18 | **A/H** |
| 19 | **A/F** |
| 20 | **D/W** |
| 21 | **S/W** |
| 22 | **V/M** |
| 23 | **L/I** |
| 24 | **L/K** |
| 25 | **L/S** |
| 26 | **G/V** |
| 27 | **T/G** |
| 28 | **exposed surface(+3)** |
| 29 | **P/P** |
| 30 | **P/Q** |
| 31 | **Com_per(H)** |
| 32 | **L/C** |
| 33 | **E/P** |
| 34 | **L/P** |
| 35 | **L/E** |
| 36 | **P/W** |
| 37 | **accessibility(+1)** |
| 38 | **T_num(C)** |
| 39 | **G/Q** |
| 40 | **disorder(-2)** |
| 41 | **I/G** |
| 42 | **Y/Q** |
| 43 | **E/A** |
| 44 | **R/T** |
| 45 | **D/L** |
| 46 | **polarity(+1)** |
| 47 | **V/H** |
| 48 | **H/G** |
| 49 | **N/F** |
| 50 | **G/G** |
| 51 | **Y/Y** |
| 52 | **flexibility(0)** |
| 53 | **W/G** |
| 54 | **disorder(+3)** |
| 55 | **hydrophilicity(0)** |
| 56 | **Y/G** |
| 57 | **Y/D** |
| 58 | **G/F** |
| 59 | **A/W** |
| 60 | **polarity(+4)** |
| 61 | **Q/P** |
| 62 | **K/T** |
| 63 | **T_num(H)** |
| 64 | **H/D** |
| 65 | **E/C** |
